# The complete mitochondrial genome of olive barb, *Systomus sarana sarana* (Hamilton, 1822) and its phylogenetic status

**DOI:** 10.1080/23802359.2017.1413319

**Published:** 2017-12-11

**Authors:** J. R. Biswal, Rajeev K. Singh, Nimisha Dutta, Abhinav Pathak, Kuldeep K. Lal, Vindhya Mohindra, R. S. Sah, J. K. Jena

**Affiliations:** a National Bureau of Plant Genetic Resources, New Delhi, India;; b National Bureau of Fish Genetic Resources, Lucknow, India;; c Indian Council of Agricultural Research, New Delhi

**Keywords:** Mitogenome, protein coding genes (PCG), next-generation sequencing, phylogeny

## Abstract

*Systomus sarana sarana*, commonly known as olive barb, is an important food and ornamental fish species with wide distribution in South Asia. Here, the complete mitogenome was sequenced on HiSeq 2500. With 16,590 nucleotides, the base composition was 32.9% (A), 26% (C), 15.4% (G) and 25.7% (T), comparable with other carps. The clustering pattern depicted the monophyly of *S. sarana sarana* with sister cyprinids.

Among cyprinids, it has been often found difficult to distinguish a species (Howes 1991) due to high morphological similarity within certain groups. Mitochondrial genes have been used potentially to resolve taxonomic issues (Khare et al. 2014; Kumar et al. 2016). However, data from complete mitogenome are more robust than individual genes for phylogenetic, phylogeographic and evolutionary applications (Miya and Nishida 2000; Wang et al. 2007; Mayden et al. 2009).

The *Systomus sarana sarana* (Family Cyprinidae) is an important food and ornamental species, and widely distributed in the Indian Rivers. It has been categorized as vulnerable due to reduction in its natural abundance on account of various pressures (Dahanukar et al. 2012). Here, we report the complete mitochondrial genome of *S. sarana sarana*, and phylogenetic status. The specimen (NBFGR/PSS525), available at ICAR-NBFGR repository, was collected from river Narmada at Dongarwada (26°46′N 77°42′E) in 2015. High-quality genomic DNA was extracted (Singh et al. 2012), amplified and sequenced on Illumina Hiseq2500. Total 3,576,250 reads were assembled and annotated on MitoAnnotator (Iwasaki et al. 2013). The phylogenetic status of *S. sarana sarana* (GenBank KU886061) was inferred using ML and NJ topologies (Tamura and Nei 1993) along with other orders.

Overall, the mitogenome has a circular molecule of 16,590 bp, containing 13 Protein Coding Genes, two rRNAs, 22 tRNAs and a control region. The arrangement of genes was typical to vertebrate/fish mitogenome. All the genes were encoded on the heavy strand except for ND6 and eight tRNA genes. The gene arrangement was consistent with the highly conserved genome architecture of cyprinids (Bej et al. 2013). The overall base composition of the mitogenome was 32.9% (A), 26.0% (C), 15.4% (G) and 25.7% (T), with A + T bias of 58.6%. The base composition of heavy strand had high A + T content (59.1%), indicating significant strand asymmetry.

The length of PCGs accounted for 68.9% of the mitogenome. Excepting COI (GTG), all of the start codons of PCGs were ATG (Satoh et al. 2016). The major non-coding region (D-loop) was 926bp and had conserved nucleotide blocks. The composition of the tRNA genes was 30.8% (A), 24.3% (C), 19.9% (G) and 25.0% (T). The ratio of non-synonymous and synonymous substitutions was <1 except for four genes (COI, COII, COIII and ATPase6). The average ratios for eight PGCs varied from 0.67 (ATP8) to 0.98 (ND5), reflecting evolution under purifying selection (Michael et al. 2006).

The phylogentic analysis was conducted for concatenated protein coding genes of cypriniformes, Siluriformes, Characiformes, Gymnotiformes and Gonorynchiformes. The phylograms (NJ and ML) produced similar topologies (Figure 1). *S. sarana sarana* formed a sister clade with other cypriniforms which is in line with the conventional classification (Nelson 2006).

**Figure 1.**
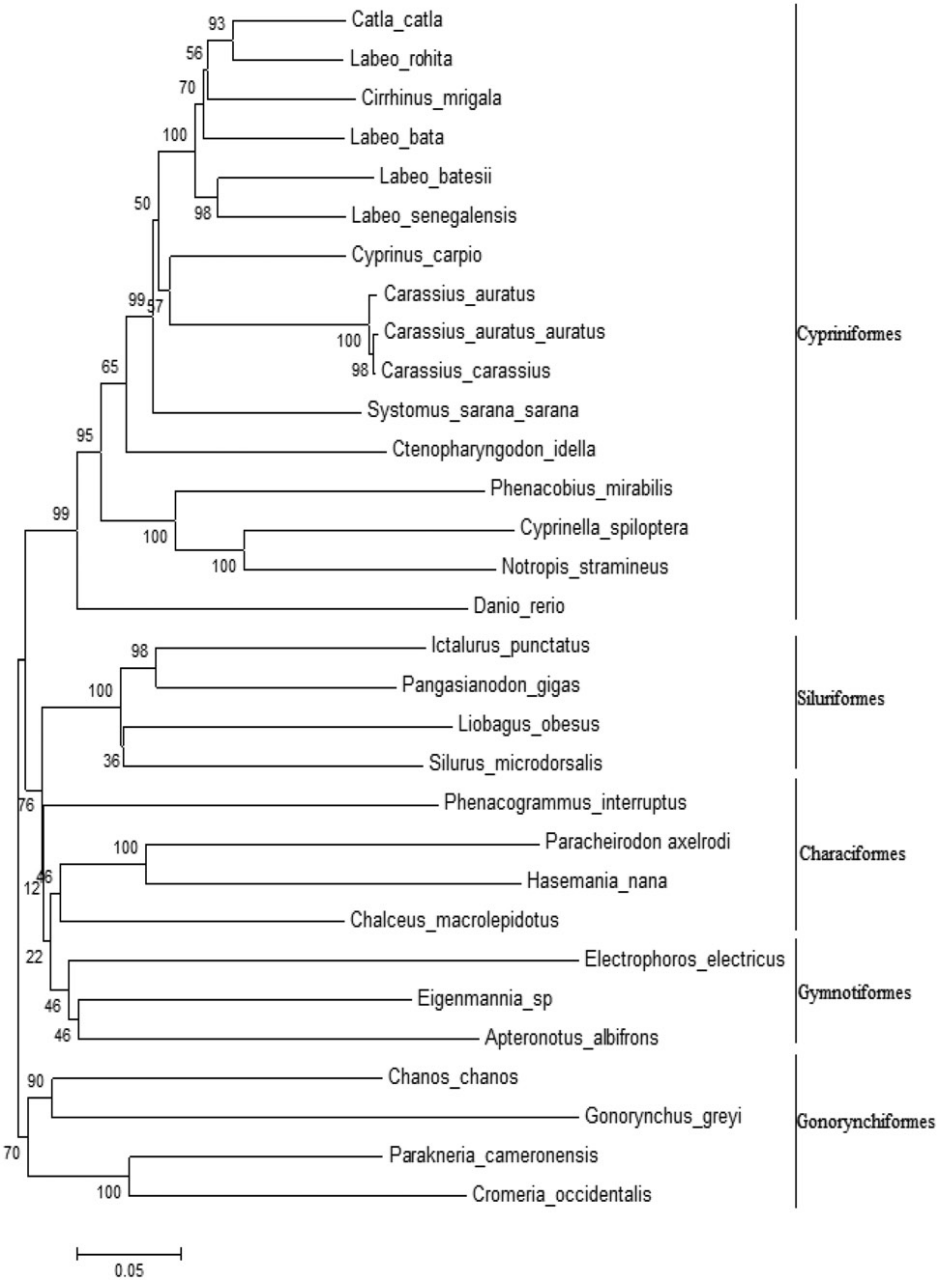
Phylogenetic tree (based on neighbour joining) from concatenated DNA sequences of mitochondrial protein coding genes.
